# Experimental and Numerical Assessment of Flexural Behavior of CFRP–Strengthened Timber Beams †

**DOI:** 10.3390/polym18010134

**Published:** 2026-01-01

**Authors:** Milot Muhaxheri, Enes Krasniqi, Naser Kabashi, Ylli Murati, Ridvan Mahmuti

**Affiliations:** Faculty of Civil Engineering, University of Prishtina, 10000 Prishtine, Kosovo; milot.muhaxheri@uni-pr.edu (M.M.); naser.kabashi@uni-pr.edu (N.K.); ylli.murati@uni-pr.edu (Y.M.); ridvanmahmuti1@gmail.com (R.M.)

**Keywords:** glulam, CFRP, flexural strengthening, experimental testing, FEM, analytical modeling

## Abstract

Glued laminated timber (glulam) is increasingly adopted as a sustainable structural material; however, its performance under bending can be limited by brittle tensile failures and variability caused by natural defects. This study examines the flexural behavior of glulam beams strengthened with externally bonded carbon fiber reinforced polymer (CFRP) sheets. A four-point bending experimental program was carried out on glulam beams with varying CFRP bonded lengths, including unreinforced control beams. The results demonstrate that CFRP reinforcement enhanced load–carrying capacity by up to 48%, increased stiffness, and shifted failure modes from brittle tension–side ruptures to more favorable compression–controlled mechanisms. A nonlinear finite element (FE) model was developed using DIANA software 10.5 to simulate the structural response of both unreinforced and CFRP–strengthened beams. The numerical model accurately reproduced the experimental load–deflection behavior, stress redistribution, and failure trends, with deviations in ultimate load prediction generally within ±16% across all reinforcement configurations. The simulations further revealed the critical influence of CFRP bonded length on stress transfer efficiency and failure mode transition, mimicking experimental observations. By integrating experimental findings with numerical simulations and simplified analytical predictions, the study demonstrates that reinforcement length and bond activation govern the effectiveness of CFRP strengthening. The proposed combined methodology provides a reliable framework for evaluating and designing CFRP strengthened glulam beams.

## 1. Introduction

Engineered timber products, such as glued laminated timber (glulam), have become increasingly prevalent in modern construction due to their favorable strength–to–weight ratio, sustainability, and adaptability. Despite these advantages, glulam beams often exhibit brittle and unpredictable behavior under bending, primarily due to tensile failures near knots, finger joints, or other natural imperfections [1,2]. These limitations have motivated growing research interest in reinforcement methods aimed at enhancing strength, stiffness, and ductility while improving the reliability of structural performance [3]. Among reinforcement strategies, externally bonded fiber–reinforced polymers (FRPs), in particular carbon FRP (CFRP), have emerged as efficient solutions due to their high tensile capacity, low density, durability, and corrosion resistance [4]. Compared with steel reinforcement, CFRP offers superior mechanical efficiency and easier installation, making it suitable for retrofitting and upgrading existing or new timber members [5].

External bonding of FRP sheets is widely recognized as an effective strengthening approach for timber elements; however, it may present aesthetic challenges in architectural applications where exposed FRP is visually intrusive. In contrast, the use of internally embedded FRP reinforcement remains relatively underexplored, despite its potential to achieve both structural improvement and better visual integration. Studies on the flexural strengthening of timber beams with FRP materials have consistently demonstrated notable mechanical improvements. Comparative tests indicate that externally bonded CFRP sheets significantly increase both stiffness and ductility under bending, highlighting their effectiveness in enhancing the structural performance of timber elements [6,7]. Prior investigations on the strengthening of timber beams with internally bonded FRP bars have shown considerable improvements in both bending capacity and ductility. In particular, the use of glass fiber reinforced polymer bars (GFRPs) embedded within the timber promoted more gradual and controlled failure modes, providing a meaningful point of comparison with the externally applied CFRP systems examined in this study, both in terms of reinforcement strategy and overall structural performance [8,9].

Several investigations have demonstrated the benefits of CFRP in strengthening timber beams. Johns and Lacroix [2] reported flexural strength gains of 40–70% with reduced variability, while Fiorelli and Dias [3] observed improved ductility with distinct two–phase failure progression. Borri et al. [4] found stiffness gains of over 20% and load capacity increases up to 60%, although prestressing showed only marginal additional benefits. Alternative approaches such as basalt FRP (BFRP) rods have also shown improvements in strength and stiffness, while enabling integrated monitoring functions [10]. Strengthening techniques have further proven effective in decayed timber, with CFRP restoring much of the original load capacity [4]. Broader studies highlight FRP’s versatility, including enhanced durability, fire resistance, and compatibility with historic structures [5,11,12]. Still, bond quality remains critical, as defects such as voids and poor adhesion can severely compromise reinforcement performance [13].

From a modeling perspective, finite element methods (FEMs) have reliably simulated FRP–reinforced timber behavior, with nonlinear analyses predicting stiffness and strength gains within 6–29% accuracy compared to experiments [2,8]. Collectively, these findings underline the potential of CFRP in both modern and historic contexts but also reveal gaps concerning reinforcement length, bonding reliability, and integrated experimental–analytical–numerical validation.

The objective of this study is to examine the structural behavior of glulam beams strengthened with externally embedded CFRP sheets of varying reinforcement lengths. The investigation integrates four components: (i) an experimental program consisting of four–point bending tests on 14 glulam beams, including unreinforced reference specimens and beams strengthened with CFRP sheets of 80, 120, 160, and 240 cm bonded lengths, to evaluate flexural strength, stiffness, and failure mechanisms; (ii) analytical assessments using simplified models assuming that plane sections remain plane, to complement the experimental results; (iii) finite element modeling (FEM) using DIANA FEA to simulate the structural response and performance of the reinforced beams; and (iv) the development of a unified experimental–analytical–numerical framework to guide design and evaluation. By systematically comparing reinforcement configurations, the study identifies the minimum effective CFRP length and validates predictive strategies, providing insights for both new construction and the retrofitting of existing timber members.

## 2. Materials and Methods

### 2.1. Materials

The experimental program involved 14 glulam beams manufactured from spruce/fir timber, a material widely used in structural applications due to its favorable strength–to–weight ratio. In this context, Fir refers to silver fir (Abies alba), a softwood species commonly employed in European structural glued laminated timber. The mechanical properties were obtained from the producer’s catalogue and verified according to EAD 130320–00–0304. The reported values include a flatwise bending strength of 32 MPa, tensile strengths of 24 MPa parallel and 0.6 MPa perpendicular to the grain, and compressive strengths of 30 MPa parallel and 4.5 MPa perpendicular to the grain. The shear and rolling shear strengths were 4.9 MPa and 1.8 MPa, respectively, while the modulus of elasticity parallel to the grain was 15,000 MPa. These results underline the strong mechanical performance of glulam along the grain and its comparatively limited resistance perpendicular to the grain.

For reinforcement, MapeWrap C UNI–AX 300 sheets were used [14]. This material consists of unidirectional high strength carbon fibers applied with epoxy resin (MapeWrap 21) [15]. The two–part epoxy adhesive (MapeWrap 21) was mixed according to the manufacturer’s specifications, with a resin to hardener ratio of 4:1 by weight, ensuring homogeneous mixing prior to application. The cured CFRP laminate exhibited an average tensile strength of 1637 MPa and a tensile modulus of 83,848 MPa, as determined from manufacturer tests according to ASTM D–3039 [16]. One CFRP layer with a nominal thickness of 0.5 mm was applied using the wet lay–up method after thorough surface preparation of the glulam beams.

### 2.2. Sample Preparation and Test Setup

**Figure 1:**
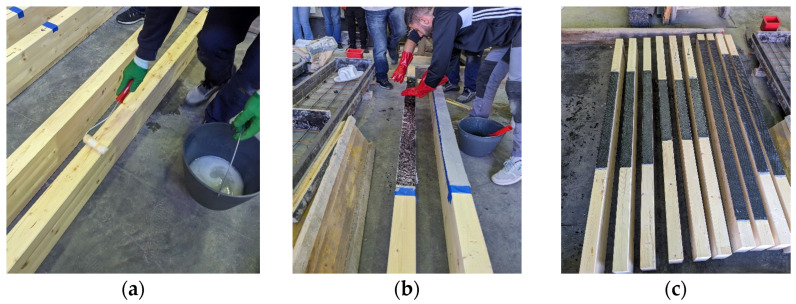
Procedure for applying CFRP sheets to glulam beams using the wet lay–up method: (**a**) application of primer layer, (**b**) placement and pressing of sheets, and (**c**) curing under controlled conditions.

The CFRP sheets were bonded to the glulam beams using the wet lay–up method, a standard approach for externally bonded FRP reinforcement in timber structures. The procedure, illustrated in Figure 1, was designed to ensure optimal adhesion and effective stress transfer between the CFRP and the timber substrate.

Surface preparation involved mechanical standing and thorough cleaning to remove contaminants and enhance adhesive penetration. A primer layer was applied to the timber surface to improve wettability and ensure compatibility between the wooden substrate and the epoxy resin.

After the primer had cured, a high–strength two–part epoxy adhesive was used to impregnate the CFRP sheets and coat the glulam surface. The CFRP was carefully positioned on the adhesive–coated timber and pressed to ensure full saturation and air void elimination. The CFRP application and curing were conducted under controlled laboratory conditions, with an ambient temperature of approximately 20 ± 2 °C and relative humidity of 50 ± 10%. After application, the specimens were left to cure for at least 7 days before testing, in accordance with the manufacturer’s recommendations.

**Figure 2:**
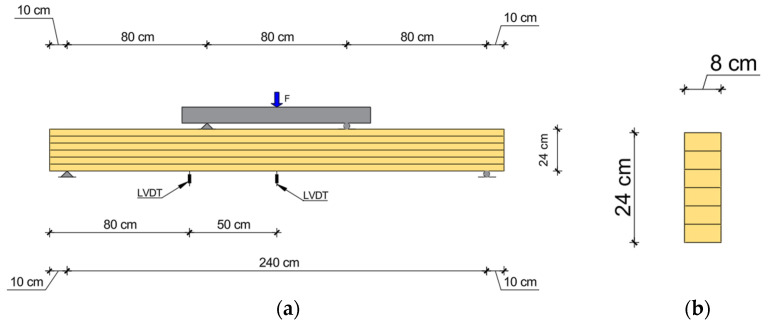
Four–point bending test setup: (**a**) load application scheme and (**b**) cross–sectional view of the glued laminated timber beam.

A four–point bending test was conducted on five groups of 14 glulam beams, following the configuration illustrated in Figure 2. Each glulam beam was composed of six spruce/fir laminations, each 4 cm thick, resulting in a cross–section of 8 cm × 24 cm and a total length of 260 cm. In the reinforced beam specimens, unidirectional CFRP sheets of varying lengths were applied along the tensile zone of the beams.

The longitudinal axis of the glulam beams corresponds to the wood grain direction, which is aligned with the beam length, consistent with standard manufacturing practice for structural glued laminated timber. A reference group of unreinforced glulam beams was tested to establish baseline performance, while four groups of CFRP–reinforced beams were fabricated to examine the influence of different reinforcement lengths on flexural behavior. This approach enabled a comprehensive evaluation of the bending response, providing valuable insights into the effectiveness of CFRP strengthening and its impact on the overall structural performance of glulam members.

All beams were tested in bending in accordance with the provisions of EN 408:2010 [17]. The specimens, with a clear span of 240 cm, were simply supported and subjected to a four–point bending configuration, as illustrated in Figure 2. The end supports were designed to mimic a pin–roller arrangement, ensuring free rotation and horizontal translation restraint at one end. Bearing plates of width 10 cm and roller bearings were placed at both the supports and loading points to prevent localized crushing and indentation.

Load was applied symmetrically at the third points of the test span using a hydraulic jack, with the applied force continuously monitored through a compression load cell. The loading protocol consisted of an initial force–controlled phase up to 10 kN, followed by monotonic displacement–controlled loading at a constant rate of 0.15 mm/s until failure. The selected loading range was based on the predicted capacity of the beams and verified through preliminary tests.

To measure the displacement of the beams, two linear variable differential transducers (LVDTs) were used. One LVDT was positioned near forces, while the second was placed at the mid–span of the beam.

## 3. Results and Discussions

### 3.1. Failure Modes

**Figure 3:**
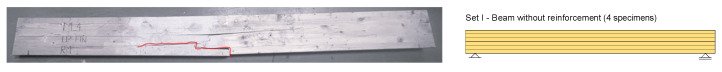
Failure mode of Set I reference beams.

The bending behavior of timber is strongly governed by the relative magnitudes of its compressive and tensile strengths. Although timber demonstrates higher tensile strength, the compressive zone tends to undergo plasticization under bending. This redistribution of stress shifts the critical demand to the tensile zone, where failure is primarily governed by stress concentration. For Set I (unreinforced beams), Figure 3, the failure mode was characterized by sudden brittle tensile rupture at the mid–span on the tension side, leading to abrupt loss of load–carrying capacity.

For the specimens corresponding to Sets II to IV, which were reinforced with CFRP strips having bond lengths of 80 cm, 120 cm, and 160 cm, respectively, the observed failure mode was predominantly associated with premature debonding phenomena. This behavior can be attributed to the development of localized stress concentrations at the termination points of the bonded reinforcement. The insufficient anchorage length limited the effective stress transfer between the timber beams and the CFRP composite, thereby promoting the initiation of interfacial cracks. Once initiated, these cracks propagated along the timber–CFRP interface, progressively undermining the adhesion and resulting in partial debonding of the reinforcement. As the debonding front advanced, the crack path deviated from the interface into the outer timber lamella, which consequently triggered localized timber fracture and ultimately led to the structural collapse of the beams.

Conversely, in the specimen where the CFRP reinforcement was extended over the entire tensile intrados, thereby providing a full bond length, the structural response and the governing failure mechanism were markedly different. In this case, the continuous bond interface enabled a more uniform stress distribution and allowed the CFRP to mobilize its full tensile capacity, delaying interfacial crack initiation. The final failure mode was characterized by lamella splitting within the timber substrate, occurring along the grain direction under the combined influence of tensile and shear stresses induced by the reinforced section. Notably, no visual signs of damage were also observed in the CFRP composite layer, indicating its active contribution to the load–carrying mechanism up to failure. This shift from interfacial debonding in the short bonded configurations to internal lamella splitting in the fully bonded configuration underscores the critical influence of bond length on both the effectiveness and the mode of failure in CFRP-strengthened timber beams.

**Figure 4:**
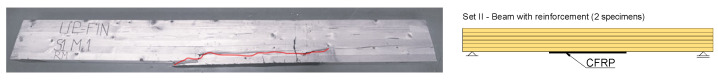
Failure mode of Set II strengthened beams with FRP bond length of 80 cm.

**Figure 5:**
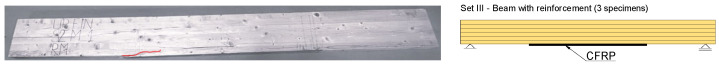
Failure mode of Set III strengthened beams with FRP bond length of 120 cm.

**Figure 6:**
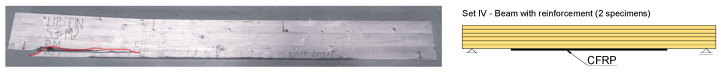
Failure mode of Set IV strengthened beams with FRP bond length of 160 cm.

**Figure 7:**
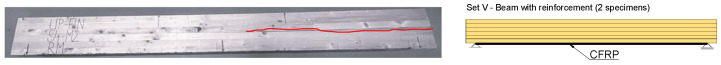
Failure mode of Set V strengthened beams with FRP bond length of 240 cm.

The experimental tests of strengthened beams showed that failure may occur with partial plasticization of the compressed zone and because of natural defects like knots followed by failure at tension or/with delamination of lamellas, as shown in Figure 4, Figure 5, Figure 6 and Figure 7.

**Figure 8:**
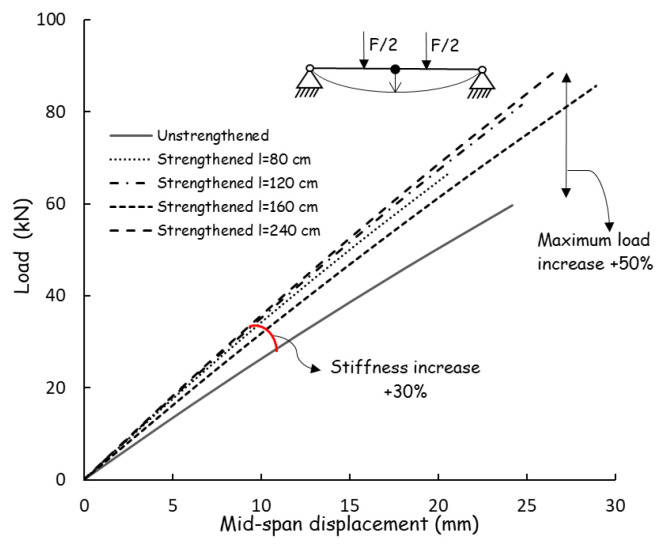
Load–displacement curves (mid–span measures of mean curves).

The average mid–span load–displacement responses of beams Set I to Set V are presented in Figure 8. The ultimate load values and the corresponding mid–span deflections of the beams are summarized in Table 1. The load–displacement response of the control beams remained nearly linear up to failure, after which a sudden crack developed at the center, leading to abrupt brittle collapse, as shown in Figure 3. The strengthened beams exhibited a marked increase in stiffness relative to the control specimens. This enhancement was evident in the load–displacement response, where the reinforced members displayed an extended linear phase under applied loading. Such behavior reflects the improved resistance to deformation and the more effective distribution of stress achieved through the contribution of the FRP reinforcement, thereby confirming the beneficial role of strengthening in enhancing the structural performance of the beams.

As observed, the mid–span deflection of the unstrengthened control beams (Set I) at failure was approximately 25 mm. In contrast, the beams strengthened with FRP reinforcement (Sets I to Set V) exhibited considerably lower mid–span displacements under the same level of applied load, with measured values ranging between 16 mm and 18 mm. This substantial reduction in deflection reflects a remarkable improvement in the beams’ behavior at the serviceability limit state (SLS), indicating that the incorporation of FRP reinforcement effectively enhanced their flexural stiffness and deformation control.

The effect of CFRP reinforcement on the load–carrying capacity is evident from the experimental results in Table 1 and the load–displacement curves in Figure 8. The unreinforced beams (Set I) failed at an average load of 66.3 kN due to brittle tensile rupture at mid–span. The introduction of CFRP resulted in a progressive increase in ultimate load with increasing bonded length. Beams with short CFRP reinforcement (Set II, 80 cm) reached an average ultimate load of 74.0 kN (+11%), while intermediate bonded lengths (Set III, 120 cm and Set IV, 160 cm) increased the capacity to 90.7 kN (+36%) and 95.2 kN (+44%), respectively. The highest load capacity was achieved with full–length reinforcement (Set V, 240 cm), reaching 98.1 kN, corresponding to an increase of approximately 48% compared to the control specimens. These results demonstrate that strengthening efficiency is strongly governed by the CFRP bonded length, as longer reinforcement enables improved stress transfer and more effective activation of the CFRP tensile capacity.

In a similar manner, CFRP reinforcement significantly influenced the flexural stiffness of the beams, as evidenced by the initial slopes of the load–displacement curves in Figure 8. The unreinforced beams (Set I) exhibited the lowest stiffness, characterized by larger mid–span deflections under increasing load. The introduction of CFRP resulted in a progressive increase in stiffness with increasing bonded length. Beams with partial reinforcement (Sets II–III) showed a noticeable stiffening effect, while specimens with longer CFRP lengths (Sets IV–V) exhibited the highest stiffness levels. Overall, the CFRP–strengthened beams demonstrated stiffness increases of approximately 20–30% compared to the control specimens. This improvement is attributed to the effective contribution of the CFRP in restraining tensile strain development in the timber, thereby reducing deflections under service–level loading and enhancing overall deformation control.

### 3.2. Numerical Modelling

Studies on the flexural behavior of FRP–reinforced wood beams have combined experimental testing with numerical simulations to evaluate structural response. The load–deflection behavior observed in reinforced specimens closely aligned with predictions from a simplified finite element model, validating its effectiveness for practical design applications [18,19].

Advanced finite element modeling of CFRP–strengthened timber elements has employed cohesive zone elements to simulate the interface behavior between CFRP and timber. The models successfully captured phenomena such as partial delamination and progressive stress transfer along the bond line, offering deeper insight into bond performance under load [8,9].

The numerical analysis was conducted using the finite element method implemented in DIANA software 10.5. The geometry and loading configurations of the numerical models were based on the experimentally tested beams to ensure consistency and accuracy in the analysis. The beam end support was represented as a roller support, restricting vertical displacement while permitting longitudinal translation, thereby replicating the experimental conditions. The timber laminations were modeled as discrete components, allowing the inclusion of their individual material properties. Consequently, the adhesive layer was not explicitly modeled due to its negligible thickness. Similarly, perfect bonding was assumed between the epoxy and timber, as well as between the epoxy and CFRP sheets, supported by prior tests demonstrating the high bond quality. To prevent stress concentration and ensure a realistic representation of contact behavior, steel plates were incorporated at the loading and support points. These plates were assumed to exhibit no slip relative to the timber, mimicking experimental observations. This approach ensured a more accurate simulation of load transfer and distribution across the beam sections. The ultimate load–carrying capacity of the beams was determined using the maximum stress criterion. Failure in the numerical model was observed at the displacement step where the computed tensile stresses in the longitudinal direction reached the tensile strength of the timber. Furthermore, due to the high tensile strength of the CFRP reinforcement and the absence of failure in the CFRP during experimental tests, the rupture of the reinforcement was not considered in the analysis.

**Figure 9:**
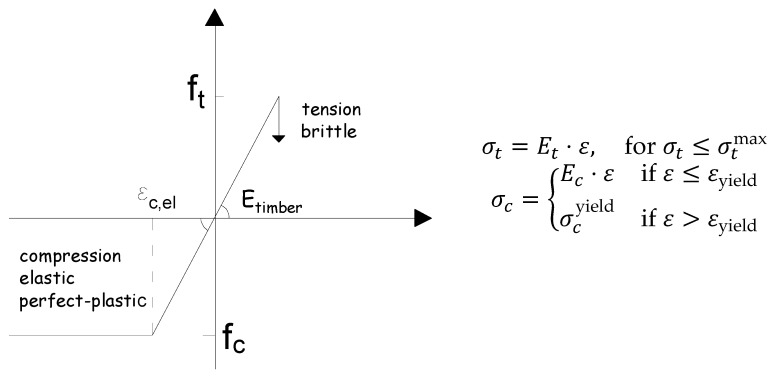
Constitutive law for timber.

The ultimate load–carrying capacity of the beams was determined using the maximum stress criterion. Failure in the model was identified at the displacement step where the computed tensile stresses in the longitudinal direction exceeded the tensile strength of the timber laminations. To maintain computational efficiency and avoid excessive complexity, timber was modeled as homogenous material. The stress–strain behavior of timber in tension was defined by a linear–elastic relationship, whereas a linear elastic–perfectly plastic relationship was employed for timber under compression. The constitutive behavior of timber is illustrated schematically in Figure 9. This approach balances accuracy with simplicity, providing a suitable representation of timber’s mechanical response within the scope of the analysis.

Timber is modeled with a Young’s modulus of 8000 N/mm^2^ and a Poisson’s ratio of 0.4. The Total Strain–Based Crack Model captures cracking under tensile stresses, while compressive behavior is defined with a strength of 30 N/mm^2^. Optional effects like creep, shrinkage, and thermal influences are excluded, focusing on static and short–term loading. FRP sheets are assigned a Young’s modulus of 83,000 N/mm^2^ and a Poisson’s ratio of 0.2, modeled using elastic–perfectly plastic behavior. The FRP–timber interface is defined with linear elastic properties: a normal stiffness of 1 N/mm^3^ (z–direction) and shear stiffness of 100 N/mm^3^ (x– and y–directions). The adopted values differ slightly from those provided in the product catalogue; however, considering the heterogeneous nature of timber, the parameters were calibrated in accordance with experimental observations.

The performance of glulam beams reinforced with externally bonded CFRP sheets was evaluated by comparing experimental and numerical results across five beam sets (I–V). Set I served as the unreinforced control group, while Sets II through V were reinforced with increasing CFRP lengths: 80 cm, 120 cm, 160 cm, and 240 cm, respectively.

A progressive improvement in load–carrying capacity was observed with increasing reinforcement length. As shown in Table 1, beams in Set V (240 cm CFRP) achieved the highest ultimate load (106.01 kN), representing an increase of approximately 48% over the average capacity of unreinforced beams in Set I (66.30 kN). The beams with partial–length reinforcement (Sets II–IV) also exhibited substantial strength gains, with load increases of 11%, 36%, and 44%, respectively. These results confirm that reinforcement length plays a critical role in maximizing the effectiveness of CFRP strengthening.

Figure 3, Figure 4, Figure 5, Figure 6 and Figure 7 illustrate the observed failure mechanisms for each reinforced beam set. In Set I (unreinforced), failure occurred through abrupt tension cracking near defects, consistent with brittle fracture in timber. In Sets II and III (partial–length reinforcement), failure initiated near the termination of the CFRP strip, suggesting stress concentration and possible interfacial debonding. Set IV exhibited mixed–mode failure, with splitting along the grain near the midspan. In contrast, Set V beams failed through compression crushing near the loading point, indicating a favorable ductile failure mechanism due to the full–span reinforcement. These observations align with the shift from tension–dominated to compression–dominated behavior as CFRP coverage increases.

**Figure 10:**
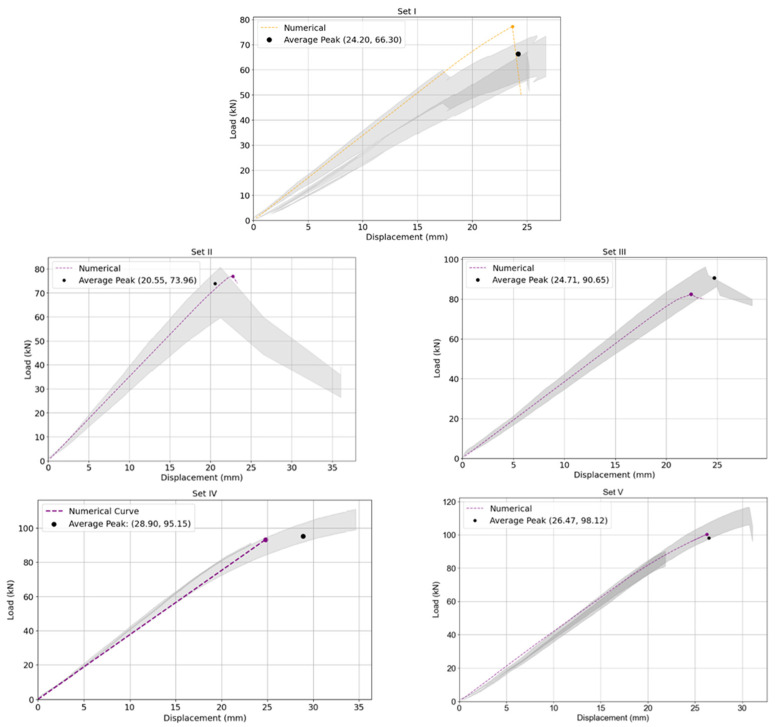
Load–deflection behavior of unreinforced (Set I) and reinforced beams (Set II, Set III, Set IV and Set V): experimental results versus numerical model predictions.

The shaded region in Figure 10 represents the experimental variability, defined as an error band corresponding to the mean and standard deviation of the measured load–displacement responses. This band accounts for the inherent scatter associated with the natural heterogeneity of glulam timber and uncertainties in material properties. Figure 10 shows that the numerical curves closely follow the experimental trends for all beam sets. The error in ultimate load prediction remained within ±16% (Table 2), validating the model’s capability to reliably simulate both stiffness and load carrying capacity under four point bending.

**Figure 11:**
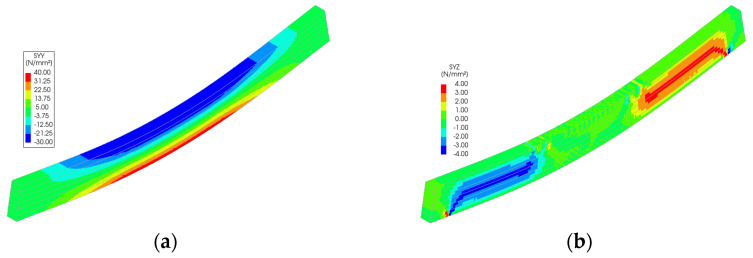
Stress distribution for Set I: (**a**) normal stress and (**b**) shear stress.

**Figure 12:**
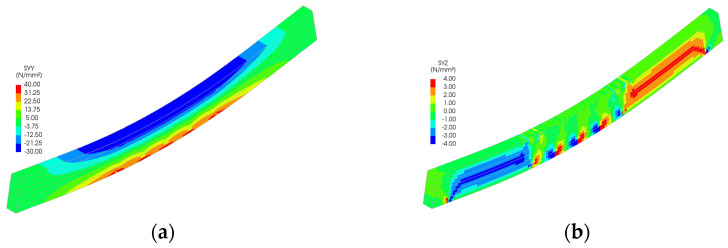
Stress distribution for Set II: (**a**) normal stress and (**b**) shear stress.

**Figure 13:**
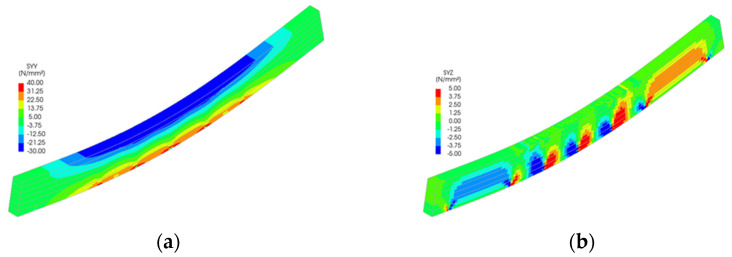
Stress distribution for Set III: (**a**) normal stress and (**b**) shear stress.

**Figure 14:**
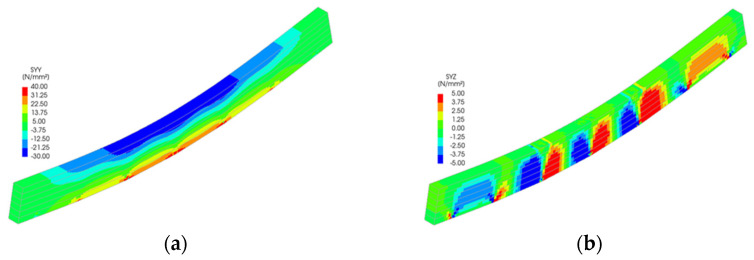
Stress distribution for Set IV: (**a**) normal stress and (**b**) shear stress.

**Figure 15:**
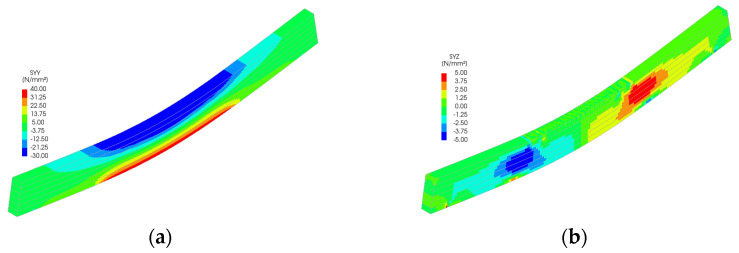
Stress distribution for Set V: (**a**) normal stress and (**b**) shear stress.

Stress distributions from the FEM simulations (Figure 11, Figure 12, Figure 13, Figure 14 and Figure 15) further support the experimental findings. In unreinforced beams, tensile stresses peaked at the tension face and initiated brittle failure. In reinforced beams, CFRP layers redistributed internal stresses, reducing tension peaks and promoting more uniform strain development. Notably, localized stress concentrations were observed near the CFRP terminations in Sets II–IV, which may explain the premature failure initiation in these specimens.

Although debonding failure was not observed explicitly in experiments, the initiation of cracks near reinforcement terminations in shorter CFRP configurations suggests that interfacial stress gradients played a role. In the FEM model, perfect bonding was assumed, which may have led to slightly conservative predictions in Sets II and III. Future studies could refine this by incorporating interface elements or cohesive zone modeling to simulate possible delamination effects.

The timber was modeled as nonlinear perfect plasticity compression response, capturing the local crushing observed near load application points. However, fracture energy and crack propagation modeling were not explicitly included in the analysis, which limits the ability to simulate splitting and shear failures observed in some sets. Still, the model’s alignment with test outcomes suggests its validity for global performance assessment.

The numerical analysis provides critical insights into the distribution of normal and shear stresses in glulam beams at the ultimate load across all test series. The shear stress in unreinforced glulam beams was observed to be uniformly distributed within the region between the support and the load application point, consistent with the theoretical shear force distribution.

For reinforced beams, the maximum shear stresses were localized near the load application points, attributed to the indentation effects at these positions. This phenomenon contributes to the combined tensile–shear failure mode observed in certain tested reinforced beams. In the beams from Sets II to V, a narrow shear stress peak was identified at the termination points of the reinforcement. However, the magnitude of this peak remained below the expected shear strength of timber, thereby ensuring the structural integrity of the reinforced system in this critical region.

These findings underscore the interplay between reinforcement and stress distribution, offering valuable implications for the design and optimization of glulam beams under loading.

### 3.3. Analytical Calculations

Analytical models provide a practical means to predict the bending behavior of glulam beams by combining simplified stress–strain laws with equilibrium conditions. When extended with the linear–elastic response of CFRP reinforcement, the model provides an efficient tool for assessing the capacity of both unreinforced and strengthened beams.

The Bazan–Buchanan analytical model provides a theoretical framework for predicting the bending response of glulam beams by combining a linear strain distribution with a nonlinear constitutive law for timber. In this approach, the section is assumed to remain plane during bending, so that the strain distribution across the depth of the beam is linear throughout the entire loading process. The stress–strain relationship of glulam is then described by an asymmetric law in which the compressive behavior is bilinear, consisting of an initial elastic phase followed by a reduced stiffness branch that accounts for post elastic plasticity and stress redistribution, whereas the tensile behavior is considered linear up to rupture, consistent with the brittle nature of timber in tension (Bazan, 1980 [20], Buchanan, 1990 [21]). These fundamental concepts have been further elaborated in later works, which provided broader design perspectives and experimental validation of timber stress–strain characteristics in structural applications (Tingley, 1997 [22]).

Through the integration of these stress–strain relationships, the position of the neutral axis can be established, enabling the prediction of bending stiffness and ultimate moment resistance. Structural failure is typically governed by rupture of the tensile fibers when their tensile strength is reached, while the bilinear compressive law allows partial redistribution in the compression zone. This model has been widely used to develop analytical predictions for unreinforced glulam members and has also provided a robust foundation for modeling timber beams strengthened with external reinforcement, particularly fiber reinforced polymer (FRP) composites. In reinforced systems, the contribution of CFRP or GFRP sheets and plates is typically incorporated by superimposing their linear–elastic stress–strain response on the nonlinear timber law, which allows enhanced moment capacity and stiffness to be captured in a consistent manner (Triantafillou & Deskovic, 1992 [23]; Gentile et al., 2002 [24]). More recently, this approach has been applied to hybrid configurations of glulam beams with CFRP laminates, confirming the ability of the Bazan–Buchanan model to reproduce experimental failure modes and load–deflection behavior (Yang et al., 2016 [25]; de la Rosa García et al., 2013 [26]). The versatility of the model lies in its capacity to incorporate the asymmetric response of timber in compression and tension, while remaining sufficiently simple to enable practical predictions of stiffness, neutral axis position, and ultimate bending resistance in both unreinforced and reinforced configurations.

**Figure 16:**
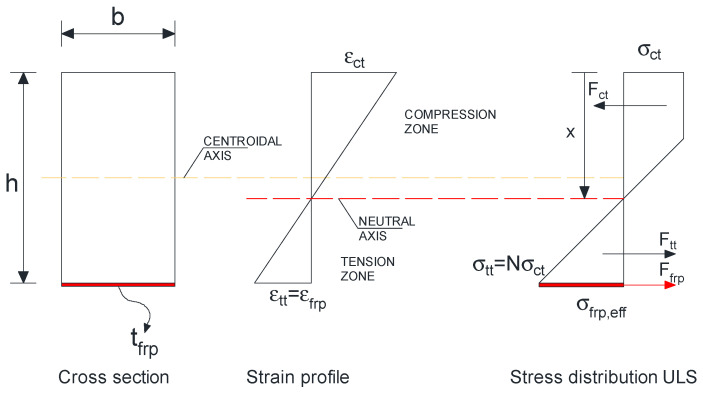
Strain and stress distributions across depth of beam at failure.

**Figure 17:**
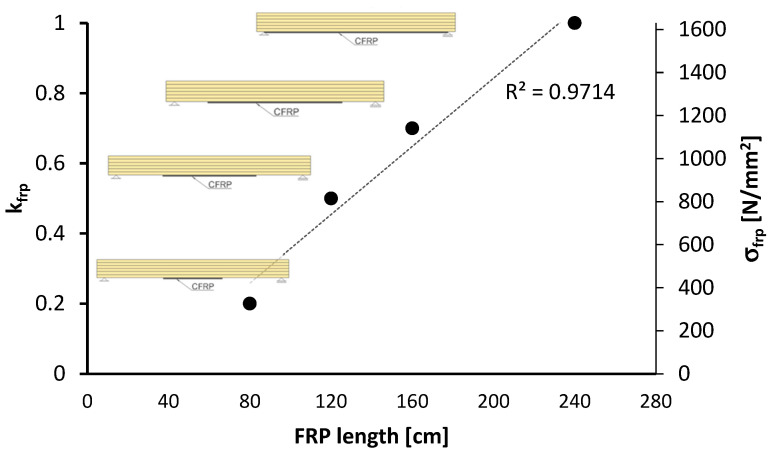
Effect of bonded CFRP length on activation.

Cross section, strain, and stress distributions at ultimate for a timber beam strengthened with a bottom CFRP laminate shown in Figure 16, together with the closed form expression for the nominal bending resistance. Plane sections are assumed to remain plane; the timber compression zone follows a bilinear (trapezoidal) stress block governed by the shape parameter N; timber tension is neglected after cracking; the CFRP behaves linear elastically up to an effective stress σ_frp,eff_ that depends on bond/anchorage. Table 3 summarizes the material parameters adopted in the analytical model. These parameters were selected to represent the mechanical behavior of the constituent materials and to ensure a consistent description of their response within the adopted analytical framework.

$$M_{u}=\sigma_{ct}\left(\frac{bh^{2}}{6}\right)\left[\frac{3N-1}{N+1}\right]+\sigma_{frp,eff}t_{frp}b_{frp}\left(h-x+\frac{t_{frp}}{2}\right)$$

where:

$$\sigma_{frp,eff}=k_{frp}\sigma_{frp}$$

k_frp_ represents bond-anchorage efficiency coefficient (Figure 17), related to the bond between FRP and the substrate, governing the effective activation of stresses in the FRP reinforcement. A value of 1.0 indicates a perfect bond condition, meaning that the full tensile strength of the FRP can be mobilized.

Relationship between bonded FRP length L_frp_ (x–axis) and the FRP activation coefficient k_frp_ (left y–axis); the corresponding effective FRP stress σ_u,frp_ is shown on the secondary y–axis in Figure 17. Each marker represents one reinforcement layout (schematics shown). The dashed line is a linear regression (R^2^ = 0.97) showing an approximately linear increase in activation with bonded length: 80–120 cm mobilizes k_frp_ = 0.2–0.55; 160 cm reaches k_frp_ = 0.7; and 240 cm approaches full activation (k_frp_ = 1.0), corresponding to σ_u,frp_ up to 1600 N/mm^2^. Adequate bonded length improves stress transfer and delays debonding, enabling near full utilization of the CFRP.

Sets SI–SV correspond to bonded lengths L_frp_ = 0, 80, 120, 160, and 240 cm, respectively. The FRP activation coefficient k_frp_ increases monotonically with anchorage length and governs the effective FRP stress according to σ_frp,eff_ = k_frp_σ_u,frp_. Using σ_u,frp_ = 1637 N/mm^2^, the resulting σ_frp,eff_ values are 0, 327.4, 818.5, 1145.9, and 1637 N/mm^2^ for SI–SV i.e., = 0%, 20%, 50%, 70%, and 100% of the nominal tensile capacity. The Table 4 provides the k_frp_ parameterization adopted in the analytical model and underscores that adequate bonded length is required to mobilize the CFRP capacity.

As M_reinf_ increases from 0 to 8.20 kNm, M_tot_ rises from 30.72 to 38.92 kNm, i.e., +5.3%, +13.4%, +18.7%, and +26.7% over the unreinforced baseline. The corresponding predicted load P_an_ increases from 76.8 to 97.3 kN. Comparison with experiments shows slight overestimation at high reinforcement (P_an_/P_ex,av_ = 1.04) and conservative predictions at lower reinforcement levels (Pan/Pex = 0.86–0.91). Overall, Table 5 indicates that increasing CFRP contribution systematically enhances capacity, and the analytical model captures the trend while becoming conservative as the CFRP effect grows.

## 4. Conclusions

This study investigated the structural performance of glulam beams reinforced with externally bonded carbon fiber reinforced polymer (CFRP) sheets through experimental testing and finite element modeling (FEM). The results confirm that CFRP reinforcement is highly effective in enhancing the flexural strength, stiffness, and failure behavior of glulam beams.

Experimental findings show that increasing the CFRP length leads to higher load carrying capacity up to 48% more than unreinforced beams and shifts the failure mode from brittle tension to more ductile compression dominated behavior. Beams with full length reinforcement (240 cm) performed the best in terms of strength and ductility, emphasizing the role of reinforcement extent in design effectiveness.

The FEM simulations successfully reproduced the load–deflection response and failure mechanisms observed in the experiments, with prediction errors generally below 14%. The numerical model accounted for key nonlinearities in timber response and internal stress redistribution induced by CFRP reinforcement.

Based on the close agreement between experimental and numerical results, the study proposes an integrated design and assessment approach that combines FEM simulations with experimentally calibrated input parameters. While not a stand–alone analytical equation, this strategy offers a practical framework for predicting the behavior of CFRP–reinforced glulam beams and informing reinforcement layout and bonding considerations.

The activation coefficient kfrp increases with bonded length and directly governs the effective FRP stress; sufficient anchorage is essential to mobilize the reinforcement. Greater CFRP contribution leads to higher total bending capacity and predicted failure load; the analytical model reproduces this trend, slightly overestimating at low reinforcement and becoming conservative as reinforcement increases. The relation between bonded length and activation is approximately linear, and improved anchorage enhances stress transfer and delays debonding, enabling near–full utilization of the CFRP.

Overall, the study underscores the effectiveness of CFRP sheets in upgrading timber structures and highlights the importance of proper reinforcement length, bonding quality, and stress distribution awareness in achieving optimal performance. These findings can support the future development of reliable strengthening techniques for both new constructions and the rehabilitation of aging timber elements.

## Figures and Tables

**Table 1:** Results from experimental bending tests.

Test Series	Number of Specimens	Average	Minimum	Maximum
Maximum load (kN)				
I	4	66.30	56.07	73.72
II	2	73.96	70.25	77.67
III	3	90.65	86.69	96.32
IV	2	95.15	91.28	99.03
V	2	98.12	90.23	106.01
Deflection at maximum load (mid–span) (mm)				
I	4	24.20	25.11	25.90
II	2	20.55	19.89	21.22
III	3	24.71	23.87	25.16
IV	2	28.90	23.19	34.61
V	2	26.47	21.84	30.62

**Table 2:** Comparison between mean experimental results and numerical predictions.

Test Series	Experimental	Numerical	Numerical/Experimental
Load (kN)			
I	66.3	77.2	1.16
II	74.0	76.96	1.04
III	90.7	82.45	0.91
IV	95.2	93.23	0.98
V	98.1	100.31	1.02
Deflection (mid–span) (mm)			
I	24.20	23.67	0.91
II	20.55	22.76	1.14
III	24.71	22.44	0.94
IV	28.90	24.80	0.75
V	26.47	26.25	0.86

**Table 3:** Material parameters used in the analytical model.

E_frp_ (MPa)	Et (MPa)	σ_c_ (MPa)	σ_t_ (MPa)	ε_c_	ε_t_	σ_u,frp_ (MPa)	N
83,848	8000	30	42	0.00375	0.00525	1637	1.4

**Table 4:** Activation of CFRP as a function of bonded length.

Set	Anchorage Length	k_frp_.	σ_u,frp,eff_ = σ_u,frp_ k_frp_
SI	0	0	0
SII	80	0.2	327.4
SIII	120	0.5	818.5
SIV	160	0.7	1145.9
SV	240	1	1637

**Table 5:** Analytical–experimental comparison of strengthening cases.

Set	M_un_ [kNm]	M_reinf_ [kNm]	M_tot_ [kNm]	P_an_ [kN]	P_ex,av_ [kN]	P_an_/P_ex,av_
I	30.72	0	30.72	76.8	66.3	0.86
II	30.72	1.64	32.36	80.90	74	0.91
III	30.72	4.10	34.82	87.05	90.7	1.04
IV	30.72	5.74	36.46	91.15	95.2	1.04
V	30.72	8.20	38.92	97.30	98.1	1.00

M_un_ = unreinforced bending resistance; M_reinf_ = CFRP contribution to bending resistance.

## Data Availability

The original contributions presented in this study are included in the article. Further inquiries can be directed to the corresponding author.
